# Evaluation of qPCR-Based Assays for Leprosy Diagnosis Directly in Clinical Specimens

**DOI:** 10.1371/journal.pntd.0001354

**Published:** 2011-10-11

**Authors:** Alejandra Nóbrega Martinez, Marcelo Ribeiro-Alves, Euzenir Nunes Sarno, Milton Ozório Moraes

**Affiliations:** 1 Laboratório de Hanseníase, Instituto Oswaldo Cruz – FIOCRUZ, Rio de Janeiro, Rio de Janeiro, Brazil; 2 CDTS, Center for Technological Development in Health, Instituto Oswaldo Cruz – FIOCRUZ, Rio de Janeiro, Rio de Janeiro, Brazil; Ege University, Turkey

## Abstract

The increased reliability and efficiency of the quantitative polymerase chain reaction (qPCR) makes it a promising tool for performing large-scale screening for infectious disease among high-risk individuals. To date, no study has evaluated the specificity and sensitivity of different qPCR assays for leprosy diagnosis using a range of clinical samples that could bias molecular results such as difficult-to-diagnose cases. In this study, qPCR assays amplifying different *M. leprae* gene targets, *sodA*, 16S rRNA, RLEP and Ag 85B were compared for leprosy differential diagnosis. qPCR assays were performed on frozen skin biopsy samples from a total of 62 patients: 21 untreated multibacillary (MB), 26 untreated paucibacillary (PB) leprosy patients, as well as 10 patients suffering from other dermatological diseases and 5 healthy donors. To develop standardized protocols and to overcome the bias resulted from using chromosome count cutoffs arbitrarily defined for different assays, decision tree classifiers were used to estimate optimum cutoffs and to evaluate the assays. As a result, we found a decreasing sensitivity for Ag 85B (66.1%), 16S rRNA (62.9%), and sodA (59.7%) optimized assay classifiers, but with similar maximum specificity for leprosy diagnosis. Conversely, the RLEP assay showed to be the most sensitive (87.1%). Moreover, RLEP assay was positive for 3 samples of patients originally not diagnosed as having leprosy, but these patients developed leprosy 5–10 years after the collection of the biopsy. In addition, 4 other samples of patients clinically classified as non-leprosy presented detectable chromosome counts in their samples by the RLEP assay suggesting that those patients either had leprosy that was misdiagnosed or a subclinical state of leprosy. Overall, these results are encouraging and suggest that RLEP assay could be useful as a sensitive diagnostic test to detect *M. leprae* infection before major clinical manifestations.

## Author Summary

Leprosy is a chronic infectious disease caused by *Mycobacterium leprae* an obligate intracellular pathogen that can infect cells in skin and nerves. Leprosy still affects 211,903 individuals per year worldwide and lead to permanent nerve injury that is generally associated with late diagnosis. The mechanisms of interaction between pathogen and the human host that leads to active disease are complex and there is no gold standard to detect *M. leprae* or host response that could early identify patients preventing severe forms of the disease, extensive nerve damage and disabilities. Thus, diagnosis relies mainly on clinical parameters and histopathological and bacteriological sometimes help to ascertain clinical form of patients. But, recently, advances in the genome of the pathogen provided extended information towards new targets to design novel genetic or immunological markers. Also novel molecular biology methods exhibiting higher sensitivity along with easy to handle apparatus based on nucleic acid detection are available. Here, we test and compare different assays for quantitative PCR (qPCR) designed to amplify specific *M. leprae* targets enriching the test sample with difficult-to-diagnose leprosy cases. Our results suggest that qPCR specially the one targeting repetitive element (RLEP) could be used to early detection of leprosy cases.

## Introduction

Leprosy is a slowly progressive spectral disease caused by *Mycobacterium leprae*, an intracellular bacterium that has a tropism for macrophages in skin and Schwann cells in peripheral nerves. In 1966, Ridley and Jopling classified a five forms spectrum disease that at one end of the spectrum is tuberculoid (TT) leprosy, where patients mount a strong cell-mediated immune response against *M. leprae* resulting in the reduction and eventual clearance of the infecting bacteria. At the other end is the lepromatous (LL), a condition characterized by highly infected and disseminated skin lesions with high levels of anti-*M. leprae* antibodies in serum and a weak cell-mediated immune response towards *M. leprae* antigens [1], [2]. In between these two polar forms, unstable borderline cases with specific clinical, immunological and pathological characteristics exist and are subdivided into borderline tuberculoid (BT), borderline borderline (BB) and borderline lepromatous (BL). In addition, among bacterial pathogens, infection of peripheral nerves is a unique property of *M. leprae* and patients can exhibit a rare form known as pure neural leprosy, PNL [3]. Moreover, very early skin lesions may be presented as relatively nonspecific perineural infiltrates in which rare acid-fast bacilli can be detected, but without sufficient infiltrates to classify them; these are called indeterminate (I). The disease is challenging to diagnose since there is no gold standard method to detect *M. leprae* or its cell components (DNA, lipids or proteins). The major difficulty in leprosy diagnosis concerns tuberculoid, indeterminate or PNL forms where acid-fast bacilli (AFB) in slit smears are very rare or absent.

Historically, one of the limitations to develop new diagnostic tests was the inability to grow *M. leprae in vitro*. Animal models such as mouse footpad [4] and armadillos [5] helped to overcome this problem and aided improvements in leprosy research. Since then, a wave of significant progress in understanding the molecular structure of *M. leprae* has been achieved including the completion of the genome sequencing of the leprosy bacillus [6]. Given that, simple and specific PCR assays for detection of small numbers of bacteria in clinical samples have been proposed. During the past 20 years, PCR methods have been developed to amplify different gene targets of *M. leprae*. These include genes encoding various *M. leprae* proteins such as the 36-kDa antigen [7], the 18-kDa antigen [8], or the 65-kDa antigen [9], Ag 85B [10], 16S rRNA [11] and the repetitive sequences (RLEP) [12]. Recently, quantitative PCR (qPCR) assays which are based on real-time quantitative fluorescence detection are replacing conventional end-point PCR in many laboratories. They have improved specificity and sensitivity for quantification of bacterial DNA or cDNA content directly in clinical samples, in addition to more rapid turnaround time, which surpasses the conventional PCR technique using gel or colorimetric detection sensitivity [10]–[13].

In fact, as much as 40–50% of cases missed by standard histology and other clinical or laboratory methods can be confirmed by the use of conventional molecular methods, and one would speculate whether qPCR would improve this rate of diagnosis [14]. However, also to be considered is the fact that PCR-based diagnosis has a considerable level of failure in confirmed leprosy cases after clinical or laboratory standards. This is probably due to variability of clinical forms, i.e. the reduced amounts of *M. leprae* among paucibacillary patients reflect the need to further optimize molecular methods. The performance of PCR assays for *M. leprae* detection, however, has only been evaluated through comparative studies: comparing two different gene targets [15] or reproduction of standardized PCR assays by few groups [8], [10], [11], [13]. Hence, standardization of the PCR assays for quality assurance of leprosy diagnosis is still lacking. Identification and establishment of standardized procedures to provide adequate clinical material, nucleic acid extraction protocol, primer target, amplicon size, PCR inhibition and control of amplicon contamination, fluorescence threshold determination, standard curve quality control, and estimated chromosome counts cutoffs will assure a more reliable and reproducible diagnosis of the disease. Assays with standardized procedures can be also of immense help for others dermatological differential diagnosis, for instance, leishmaniasis, cutaneous tuberculosis, sarcoidosis, where pathological examination is inconclusive.

In the present study, we have developed and evaluated decision tree classifiers [16] from absolute chromosome count estimates based on standard curves built from qPCR assays for leprosy diagnosis, which account for biological and clinical heterogeneity (TT vs LL). Four previously described TaqMan® qPCR assays were compared for the identification of *M. leprae* in 62 skin biopsies from patients diagnosed with leprosy, skin biopsies from patients initially suspect of having leprosy, or healthy skin of non-leprosy subjects. The comparisons were made based on the following gene targets: Ag 85B [10], *sodA* and 16S rRNA [12] and RLEP [13] and a confirmatory diagnosis from patients previously diagnosed by a committee of experts (pathologists and dermatologists) based on clinical and laboratorial tests at the outpatient unit of the Oswaldo Cruz Institute, Fiocruz. We intentionally include a higher proportion of paucibacillary samples, especially the cases where rarely *M. leprae* (DNA or bacilli) is detected. These cases correspond to the indeterminate, pure neural and tuberculoid forms, i.e., exactly when the leprosy diagnosis is more challenging. Thus, the intent here was to identify and select the most sensitive and specific PCR assay useful for differential and early diagnosis of leprosy.

## Materials and Methods

### Objectives

The main goal of this study was to comparatively evaluate four different TaqMan® qPCR assays for leprosy diagnosis using a range of clinical samples that could bias the results. To develop standardized protocols and to overcome the bias resulted from arbitrarily analysis of the assays, we propose the use of decision tree classifiers to estimate optimum cutoffs. The following gene targets were used to determine the presence and levels of *M. leprae*: Ag 85A, *sodA* and 16S rRNA and RLEP.

### Collection and processing of clinical material

Punch skin biopsy (6 mm^3^) specimens from patients were obtained at the outpatient unit of the Oswaldo Cruz Institute, Fiocruz, Rio de Janeiro, Brazil. The patients were classified clinically, bacteriologically, and histopathologically, according to the Ridley-Jopling scale (R&J) [1]. A total of 62 skin biopsies were included in this study (Table 1 and Table S1). Multibacillary (MB) patients (LL, BL and BB) had bacteriological indexes (BI) ranging from +1 to +5.5 while all paucibacillary (PB) patients (including BT, I, and PNL forms) had negative BI. The logarithmic index of bacilli in the biopsy (LBI) [17] of MB patients was also evaluated and ranged from 1 to 6. Ten skin biopsy specimens from individuals that had suspicion of leprosy, but were further evaluated and clinically diagnosed with other skin diseases and five biopsies from normal healthy skin were also included as endemic controls (Table S1). Before the study was undertaken, all individuals participating in this study were informed of the purpose of the study, and written consent was obtained from all participants. The study was approved by the FIOCRUZ Ethical Committee.

**Table 1:** Summary description of skin biopsy specimens used in the study.

Patients	Number of skin biopsies	Clinical Forms*						
		PB				MB		
		I	PNL	TT	BT	BB	BL	LL
**Non leprosy**	15	-	-	-	-	-	-	-
**leprosy**	47	12	1	2	11	5	5	11

*according to the Ridley and Jopling classification. PB-paucibacillary, MB- multibacillary, I-indeterminate, PNL – pure neural leprosy, TT- tuberculoid, BT- borderline tuberculoid, BB- borderline; BL- borderline lepromatous; LL- lepromatous.

### DNA extraction from skin biopsy specimens

DNA was extracted from half of the 6 mm^3^ skin biopsy specimens using proteinase K digestion as described elsewhere [10] with modifications. Briefly, biopsy specimens were thawed at room temperature, minced and digested for 12 h at 60°C with proteinase K (300 µg/ml) in 100 mM Tris-HCl (pH 7.4), 150 mM NaCl, and 10 mM EDTA (pH 8.0). In order to help break *M. leprae* cell wall, biopsy samples were then added to FastRNA® Blue tubes and homogenized twice in the FastPrep® FP 24 instrument (Qbiogene, Carlsbad, CA, USA) at a speed setting of 6.5 for 45 sec×2 with 5 min rest between homogenizations. The homogenates were extracted with phenol∶chloroform∶isoamyl alcohol. DNA was precipitated with isopropanol, washed in 70% ethanol, dried at room temperature, and resuspended in approximately 30 µl RNase Free H_2_O.

### Quantitative Polimerase Chain Reaction (qPCR)

The levels of *M. leprae* Ag 85B [10], *sodA* [12], 16S rRNA [12], and RLEP [13] in skin biopsy specimens were estimated using TaqMan® qPCR amplification. Purified total DNA (200 ng) in 2 µl were added to a total PCR reaction volume of 25 µl containing TaqMan® 2× master mix, 500 nM of each primer and 100 nM of each probe for *sodA* or 16S rRNA PCR assays, 200 nM of each primer and 100 nM of the probe for RLEP PCR assay or 300 nM each primer and 100 nM probe for 85B PCR assay. Reaction mixtures were prepared in duplicates and subjected to 50°C for 2 min, 95°C for 10 min, and 40 cycles of 95°C for 15 sec and 60°C for 1 min using a 7000 real-time PCR system (Applied BioSystems, Carlsbad, CA, USA). Fluorescent accumulation data for skin biopsies specimens were analyzed by the ABI PRISM 7000 Sequence Detection System software (Applied Biosystems, Carlsbad, CA, USA), and *ΔRn* values extracted. Cycle threshold (C_t_) determination from *ΔRn* data was conducted in the open source software R version 2.9.1 (available at http://www.R-project.org/).

### Standard curves for qPCR assays

Standard curves were generated for each qPCR assay using five titration curves with 10-fold dilutions of purified *M. leprae* DNA from nude mouse footpads (kindly provided by Dr. Phillip Suffys) with doses ranging from 1 ng to 10 fg. Fluorescent accumulation data for titration curves and were analyzed by the ABI PRISM 7000 Sequence Detection System software (Applied BioAssays, Carlsbad, CA, USA), and *ΔRn* values extracted. Cycle threshold (C_t_) determination from *ΔRn* data was also conducted in the open source software R version 2.9.1. Ct values were plotted against input log-doses (base 10) and standard curves determined by a linear regression, and the coefficient of determination (*R^2^*) used as quality control. Then, the fitted standard curves were used to estimate *M. leprae* chromosome counts for skin biopsies specimens, considering one *M. leprae* genome to be equivalent to 3 fg [12].

### Decision Trees building and evaluation

Classification trees for this project used the open source software R version 2.9.1 implementation of the Quinlan's C4.5 algorithm [16] available in the packages ‘rpart’ and ‘caret’ (available at http://cran.r-project.org/web/packages/), for training and evaluation, respectively. Decision tree classifiers were trained with the set of estimated *M. leprae* chromosome counts derived from standard curves from the four different TaqMan® qPCR diagnostic assays previously described, and classes were assigned as *C_1_* and *C_2_* indicating that the skin biopsy specimen belongs to a confirmed leprosy patient or to a non-leprosy patient, respectively. Training parameters included: (1) prior probabilities for classes *C_1_* and *C_2_* equals 0.5; (2) 20, as the minimum number of observations that must exist in a node, in order for a split to be attempted; (3) 10, as the minimum number of observations in any terminal leaf node; and (4) Gini impurity as a measure of how often a randomly chosen element from the set would be incorrectly labeled if it were randomly labeled according to the distribution of labels in the subset. A series of 5 tree classifiers were built with different compositions of the input data. Four classifiers were built with single attributes, given by the chromosome counts from each diagnosis assay, while the fifth tree were built with all 4 attributes available, and then pruned in order to minimize the expected 10-fold cross-validation prediction accuracy. Pruning included a complexity parameter of 0.05, informing the program that any split which does not improve the fit by 0.05 will likely be pruned off by cross-validation, and that hence the algorithm need not pursue it. Also, performances of the built tree classifier were estimated by its specificity, sensitivity, and by the trapezoidal approximation of the area under the receiver operating characteristic (ROC) curve (AUC), a graphical plot of the sensitivity, or true positive rate (sensitivity), vs. false positive rate (1−specificity), for the decision tree classifier as its discrimination cutoffs were varied. AUC can be seen as a measure of commitment between sensitivity and specificity. R code is available under request to authors.

## Results

### Building of qPCR assays for leprosy diagnosis in clinical samples

**Figure 1:**
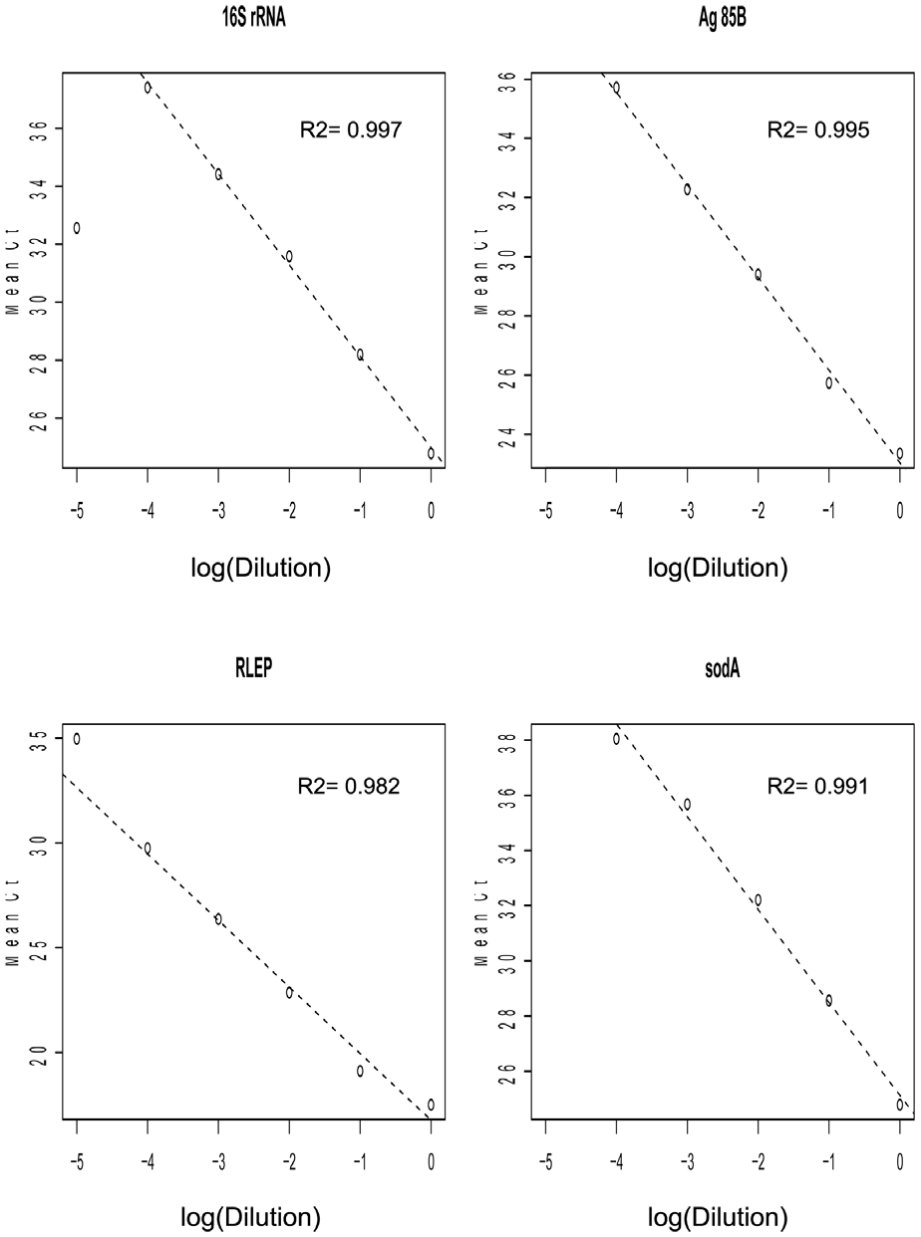
Standard curves of the amplification of 16S rRNA, Ag 85B, RLEP, and sodA targets in *M. leprae*. A range from 1 ng to 10 fg using *M.leprae* DNA for each qPCR assay was performed.

**Figure 2:**
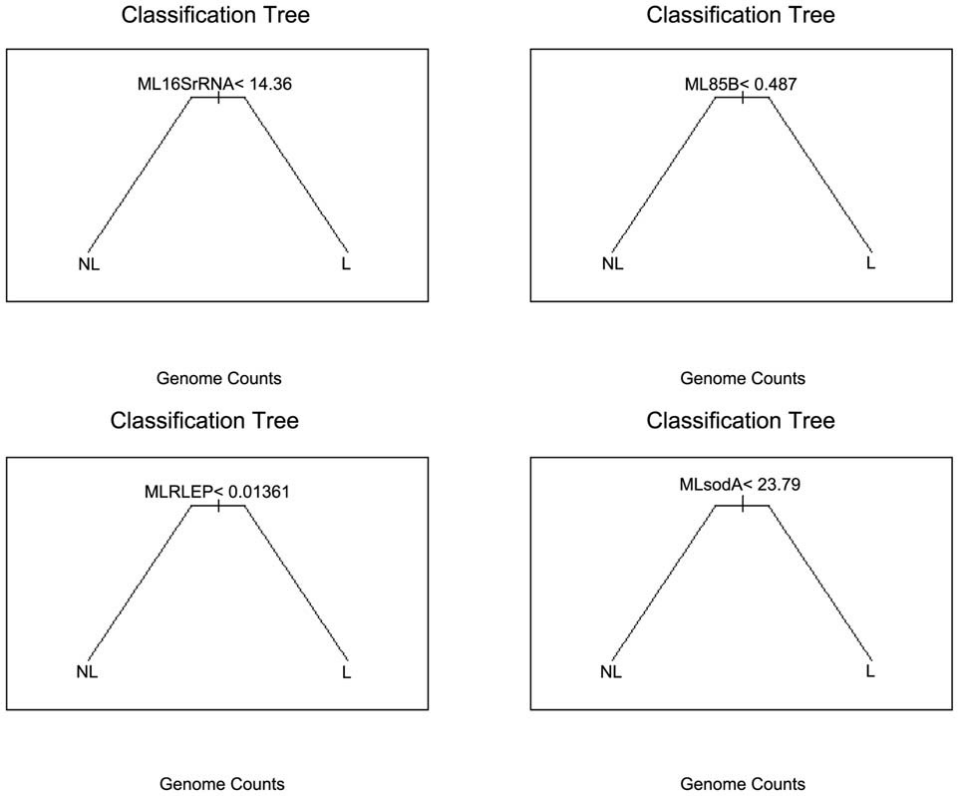
Classification trees partitions based on *M. leprae* chromosome counts. qPCR for 16S rRNA, 85B, RLEP and sodA assays into leprosy (L) or non-leprosy (NL) diagnosis. We have found different optimum chromosome count (genome counts) cutoffs for predicting leprosy, approximately greater than or equal to 14.36, 0.49, 0.01 and 23.79, respectively.

Standard curves were built from the linear regression of C_t_ value estimates from five titration curves with replication with 10-fold dilutions of purified *M. leprae* DNA ranging from 1 ng to 10 fg for each qPCR assay (Table 2; Figure 1). Quality control was guaranteed by the high coefficient of determination values achieved, ranging from 98.2% to 99.7% (Figure 1). Moreover, to control any possible PCR inhibition, TaqMan® qPCR targeting the human TNF gene was also performed and all samples tested positive (data not shown) indicating no inhibition. Optimal fluorescence thresholds were chosen based on the common practice that it should be positioned on the lower half of the fluorescence accumulation curves plot from the 10-fold dilutions and was used both to calculate the cycle thresholds (C_t_) for standard curves fitting and to calculate C_t_ for a total of 62 skin biopsy samples, 47 from untreated leprosy patients and 15 from patients suffering from other dermatological diseases and healthy donors. Considering the relation of one *M. leprae* genome at each 3 fg dilution [10], doses estimated for each skin biopsies specimen from all qPCR assays were converted to chromosome counts and used as input to train classification trees for optimization of qPCR specific chromosome counts cutoffs used in leprosy diagnosis (Figure 2). The classification trees were also used to estimate generalized errors that should be expected when using these qPCR assays for leprosy diagnosis in unforeseen samples, not used in tree fitting. These generalized errors were estimated by the 10-fold cross-validation prediction accuracy (10-fAcc.), where data is partitioned in 10 parts according to the original class distribution and at each run 9 parts are used for training and one part is used for the estimation of the test accuracy, leading to a mean value after the end of the 10 independent runs. Besides the 10-fAcc., we also report specificity, clinical sensitivity and the AUC for each of the four qPCR assays (Table 3).

**Table 2:** Standard curves parameters and results for qPCR assays of *M. leprae* DNA.

Assay	Fluorescence Threshold[1]	Linear Coefficients[2]		R^2^ [3]	Amplification
		Slope	Intercept		Efficiency
**RLEP**	0.075	−3.17	16.78	0.99	2.07
**16S rRNA**	0.161	−3.15	24.99	1	2.08
**Soda**	0.229	−3.36	25.13	0.99	1.98
**Ag 85B**	0.143	−3.13	23.04	1	2.09

[1] Fluorescence values used as threshold for determining Ct values;

[2] Linear coefficients from a line: y = *a*+*b*x+*ε*, where *a* is the intercept, *b* is the slope and *ε* is the fitting error;

[3] Standard curves coefficient of determination.

**Table 3:** Summary of the results of the decision tree classifier for leprosy diagnosis.

Assay	Mean 10-fAcc.(CI 95%)[1]	Sensitivity	Specificity	AUC[2]
**RLEP**	0.871 [0.762, 0.943]	0.915	0.733	0.824
**16S rRNA**	0.629 [0.497, 0.748]	0.511	1	0.756
**sodA**	0.597 [0.464, 0.720]	0.468	1	0.734
**Ag 85B**	0.661 [0.530, 0.777]	0.553	1	0.777
**All**	0.871 [0.762, 0.943]	0.915	0.733	0.824

[1] Mean 10-fold cross-validation accuracy with 95% confidence interval;

[2] Approximate area under ROC curve.

### Evaluation of qPCR assays for leprosy diagnosis in clinical samples

Based on the standard curves for the four assays, using C_t_ values obtained from 62 skin biopsy samples using the same fluorescence thresholds (Tables 2 and 4), we estimated the chromosome counts for each sample. Patients and control groups were classified according to clinical, bacteriological and histopathological criteria by experienced dermatologists and pathologists. Patients' names from other dermatological diseases were searched at disease surveillance and control database of Brazilian publicly-funded health care system, SUS (Portuguese for Unified Health System). Indeed, three out of seven patients initially classified as controls had a confirmation of leprosy after databank search (5–10 years after biopsy collection). Therefore, those patients were reanalyzed histologically and reclassified according to R&J and then, both chromosome counts and diagnostic labels were used in the evaluation of four different qPCR assays for leprosy diagnosis. We found the estimated chromosome counts for each assay to be in agreement with Ridley and Jopling scale for MB leprosy forms (Figure S1). As a result of the evaluation, similar specificity and sensitivity were found for all four assays with RLEP assay being more sensitive (Table 3), with optimum chromosome counts cutoffs for leprosy diagnosis estimated as greater than or equal to 0.01, 14.36, 23.79 and 0.49 for RLEP, 16S rRNA, *sodA* and Ag 85B, respectively. The fact that the expected mean classification accuracy in distinguishing leprosy patients from non-leprosy with different qPCR assays ranges from 59.7 to 87.1% is not surprising due to the very low number of bacilli expected in I and BT (negative BI patients) cases, which comprised 55.31% (26/47) of leprosy samples in the dataset. As a result, since the highest proportions of the leprosy cases belonged to indeterminate (25.53%; 12/47), borderline tuberculoid (23.4%; 11/47), in addition to tuberculoid (TT) and pure neural, i.e. PNL, (27.65%; 13/47) forms (Table 1; Table S1), prediction of patients was expected to be extremely difficult. However, this is more in accordance to the challenging leprosy diagnostics found in day-to-day practice of a typical outpatient unit, which is also in accordance with our aim to validate *M. leprae* DNA detection toward the situations where PCR could be really useful. These results show that TaqMan® qPCR targeting the multicopy RLEP sequence outperforms other single targeting TaqMan® qPCR assays tested, which in turn have comparable detection, for assessing clinical samples. In addition, the RLEP qPCR assay proved to be more sensitive than others assays and has also a larger approximated area under ROC curve (Table 3), which shows a better commitment between sensitivity and specificity for this assay. Moreover, the performance of the classifier trained with the chromosome count estimated by all 4 assays did not improve the ability to correctly distinguish leprosy patients with that of the RLEP qPCR assay alone (Table 3).

**Table 4:** PCR positivity for different real-time PCR assays.

Clinical Form	Total number of biopsies	sodA(%) positivity	16S (%) positivity	RLEP (%) positivity	85B (%) positivity
**BB**	5	4 (80)	5 (100)	5 (100)	5 (100)
**BL**	5	5 (100)	5 (100)	5 (100)	5 (100)
**LL**	11	11 (100)	11 (100)	11 (100)	10 (90.9)
**BT**	11[a]	2 (18.2)	2 (18.2)	6 (54.5)	4 (36.4)
**I**	12[b]	0	1 (8.3)	9 (75)	2 (16.7)
**NP/TT**	3	0	0	2 (66.7)	0
**Skin from healthy donor**	5	0	0	0	0
**Other dermatological diseases**	10	0	0	4	0

[a],[b] 1 BT (borderline tuberculoid) and 2 I (indeterminate) patients were initially classified as controls but confirmation of leprosy was done only after databank search (5–10 years after biopsy collection). Then, biopsies were reanalyzed histologically and also reclassified as patients according to R&J classification.

## Discussion

This is the first systematic PCR comparative evaluation using different real-time assays for detection of *M. leprae* DNA in skin biopsies from patients of different clinical forms of leprosy as well as non-leprosy dermatological diseases and healthy individuals. Our previous work [10] indicated that Ag 85B assay was specific as it did not amplify any DNA in healthy skin biopsies. In fact, none of the assays used in this study have shown amplification for healthy individuals. The introduction in this study of patients who had suspicion of leprosy like chronic dermatitis, capillaritis, cutaneous mucinosis, bacterial erythema, leukocytoclastic vasculitis and folliculitis increased the complexity of the differential diagnosis, which justifies the lower estimated sensitivities found as compared to previous studies [10], [15], [18], [19]. Four samples classified as leprosy by RLEP assay presented detectable chromosome counts ranging from 0.04 to 3.16. These putative false positive results have to be interpreted very carefully as those patients had a previous suspicion of leprosy. Recently, Goulart and colleagues [20] described that among all nucleic acid markers for leprosy diagnosis in the literature, three presented higher sensitivity and specificity (RLEP, Ag 85B and 16S rRNA). Indeed, our results demonstrated that RLEP qPCR assay could be used to improve patient detection due to its high sensitivity/confidence (100/88.9% for MB patients and 84.6/80.5% for PB patients), although the specificity of 73.3% has to be taken into consideration. As stated before, it is possible that the four patients clinically and histologically classified as non-leprosy had in fact leprosy that was misdiagnosed at the time or even a subclinical state of leprosy, especially because the area in Rio de Janeiro, where those people were diagnosed, is highly endemic. It is common to observe a very long incubation period to leprosy outcome and subclinical stages with dormant *M.leprae* within granulomas are likely to occur [21]. Nevertheless, those patients were followed up for up to 10 years and did not develop the disease. Thus, PCR positivity might indeed represent carriage of bacilli or subclinical infection, which does not indicate by itself the evolution towards the disease. An opposite speculation is that the repetitive sequence (RLEP) is highly conserved and as a result, many homologous sequences may be present in other environmental *Mycobacterium* species that have not been thoroughly investigated, generating false positive results, as reported for the *M. tuberculosis* IS6110 marker elsewhere [22], [23]. If this last hypothesis is true, then the use of a single copy gene such as the Ag 85B is favored and seems to be a more promising candidate for PCR-based diagnosis since it presented the highest confidence (55.3%) considering the PB patients as well and compared to all three others qPCR assays and 100% specificity.

It is feasible that the two most challenge features when implementing a diagnostic assay based on qPCR by absolute quantification are: the choices of the chromosome count cutoff and its generalization error, or the accuracy of the assay for classifying a new sample. In this study, these challenges were solved by using decision tree classifiers. As result, in different qPCR assays evaluated, namely RLEP, Ag 85B, *sodA* and 16S rRNA, we have found different optimum chromosome count cutoffs for predicting leprosy, approximately greater than or equal to 0.01, 0.49, 23.79 and 14.36, respectively (Figure 1; Table S1). Not surprisingly, assays with higher chromosome count cutoffs had lower sensitivity than those with lower cutoff, but with a decrease in its specificity (Table 3), which, in turn, indicates an adequate classification fit to the training data. Also, assays with lower chromosome count cutoffs had lower generalization error (10-fAcc.; Table 3), and so have a higher confidence while predicting leprosy in undetermined samples.

The use of any classification assay has limitations, especially those that oversimplify a complex disease such as leprosy. In the absence of commonly accepted reference procedures the choice of data processing is currently at the discretion of the researcher. Also, since there is a shortage of publications discussing the comparison of different DNA-based PCR assays for the detection and enumeration of *M. leprae* this study provides a comparison of four qPCR assays previously standardized and published in the literature. Notwithstanding, an external quality assurance study on diagnostic proficiency, which includes certifying and publishing the results in a comparative and anonymous manner for leprosy research would be ideal. A multicenter study with blinded samples is essential.

Other laboratory tests, such as the ELISA for anti-phenolic glycolipid I (PGL-I) IgM antibodies, are non-invasive and useful as an additional aid in diagnosis [24], [25]. The presence of anti-PGL-I antibodies is known to correlate with the bacterial load [26], and thus offers a further refinement of the WHO classification into patients with high and low bacterial loads. However, PCR has been proven to be more sensitive than serological tests, especially in paucibacillary patients [25], [27]. Also, due to the complex and varying immune responses that characterize leprosy spectrum, improved serological tests are hard to achieve and still needed. Hence, even though serological tests may have epidemiological relevance, the higher sensitivity of the PCR technique makes it a more robust tool for leprosy diagnosis. Obviously, that it should be taken into consideration that introduction of qPCR in routine diagnosis of leprosy is not easy since it is an expensive and laborious technique. But, it is likely that qPCR will become cheaper and soon surpass the conventional technique by becoming the gold standard laboratory test for leprosy diagnosis.

In tuberculosis, assays based on genexpert technology are currently in use for detection of active disease and resistance [28]. In leprosy, a rapid and early diagnostic tool, possibly based on qPCR, is still needed. The histopathologic and immunologic features of indeterminate cases suggest that it is an early form of the disease. Surprisingly, RLEP PCR assay correctly identified 75% of those patients suggesting that this can be used as a sensitive diagnostic test to detect *M. leprae* infection before major clinical manifestations. Recently, Banarjee and coworkers [29] presented that the use of PCR positivity in a follow-up of patients' contacts could predict the outcome of leprosy in 20% of the individuals suggesting that a qPCR would help detect and quantify *M. leprae* in these patients indicating chemoprophylaxis of contacts when needed. Hence, we believe that quantitation of *M. leprae* bacterial loads using RLEP qPCR will contribute to understanding mechanisms as well as being clinically important in targeting follow-up of high risk individuals and in the development of strategies for early detection and prevention.

## Supporting Information

Figure S1 **Quantitation of** ***M. leprae*** **chromosome counts as a function of the clinical form of leprosy.** I-indeterminate, PNL – pure neural leprosy, TT- tuberculoid, BT borderline tuberculoid, BB borderline; BL- borderline lepromatous; LL- lepromatous.
(DOC)

Table S1 **Analysis of** ***M. leprae*** **DNA detection for different real-time PCR assays in leprosy.** DNA samples from 62 samples of leprosy patients from different clinical forms (treated and untreated), and also patients from other dermatological conditions and normal skin from healthy donors were tested for 16S, 85B, RLEP, and sodA PCR assays.
(PDF)
