# Supplementary material for: Evaluation of qPCR-Based Assays for Leprosy Diagnosis Directly in Clinical Specimens
Source: PLoS Negl Trop Dis. 2011 Oct 11;5(10):e1354. doi: 10.1371/journal.pntd.0001354 (PMC3191141; doi:10.1371/journal.pntd.0001354)
Supplement: Figure S1 — Quantitation of M. leprae chromosome counts as a function of the clinical form of leprosy. I-indeterminate, PNL – pure neural leprosy, TT- tuberculoid, BT borderline tuberculoid, BB borderline; BL- borderline lepromatous; LL- lepromatous. (DOC) [file pntd.0001354.s001.doc]

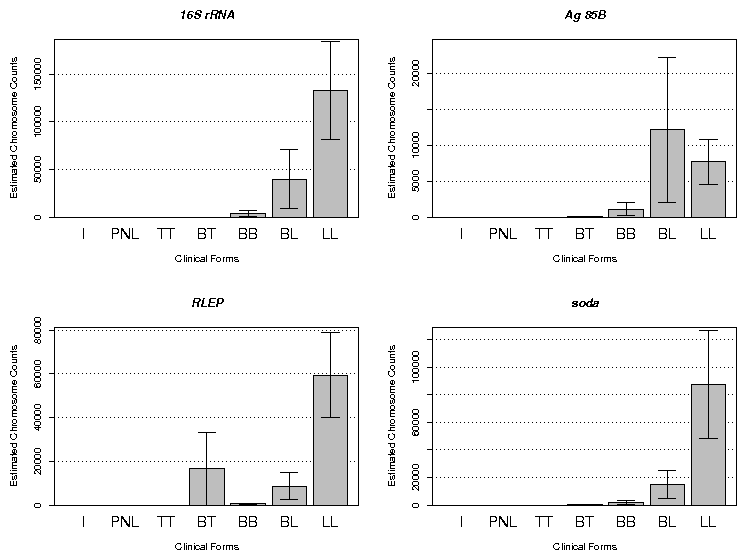


Figure S1. Quantitation of *M. leprae* chromosome counts as a function of the clinical form of leprosy. I-indeterminate, PNL – pure neural leprosy, TT- tuberculoid, BT borderline tuberculoid, BB borderline; BL- borderline lepromatous; LL- lepromatous.
