# Supplementary material for: Evaluation of qPCR-Based Assays for Leprosy Diagnosis Directly in Clinical Specimens
Source: PLoS Negl Trop Dis. 2011 Oct 11;5(10):e1354. doi: 10.1371/journal.pntd.0001354 (PMC3191141; doi:10.1371/journal.pntd.0001354)
Supplement: Table S1 — Analysis of M. leprae DNA detection for different real-time PCR assays in leprosy. DNA samples from 62 samples of leprosy patients from different clinical forms (treated and untreated), and also patients from other dermatological conditions and normal skin from healthy donors were tested for 16S, 85B, RLEP, and sodA PCR assays. (PDF) [file pntd.0001354.s002.pdf]

Table S1. Analysis of *M. leprae* DNA detection for different real-time PCR assays in leprosy.
DNA samples from 62 samples of leprosy patients from different clinical forms (treated and untreated),
and also patients from other dermatological conditions and normal skin from healthy donors were tested for 16S, 85B, RLEP, and sodA PCR assays

Multibacillary patients

Biopsy Number	Observations	Clinical Form	Ct 16S rRNA	Pred. Class 16S	Ct 85B	Pred. Class 85B	Ct RLEP	Pred. Class RLEP	Ct sodA	Pred. Class sodA
1	RR	BB	32,34	L	35,68	L	25,42	L	33,11	L
32		BB	36,87	L	38,95	L	28,11	L	NA	NL
81		BB	28,42	L	28,13	L	23,19	L	29,76	L
83	RR	BB	32,44	L	31,68	L	26,00	L	33,00	L
86	RR	BB	33,77	L	31,31	L	27,81	L	35,53	L
2	Regression/ not detectable bacilli/ ENL	BL	35,59	L	39,66	L	29,03	L	35,54	L
13	RR	BL	32,33	L	35,18	L	25,47	L	31,88	L
33		BL	29,73	L	33,19	L	23,41	L	35,64	L
84		BL	27,58	L	27,31	L	21,24	L	28,44	L
85	RR	BL	25,28	L	24,88	L	19,25	L	27,07	L
4		LL	29,50	L	32,02	L	22,71	L	31,47	L
6		LL	28,57	L	32,21	L	21,80	L	33,80	L
9		LL	27,16	L	28,79	L	20,26	L	26,64	L

15		LL	28,35	L	30,08	L	21,36	L	28,24	L
18		LL	27,26	L	28,80	L	19,89	L	26,43	L
21		LL	24,30	L	26,21	L	17,31	L	25,24	L
23		LL	23,78	L	25,50	L	16,89	L	23,97	L
25		LL	36,66	L	NA	NL	29,35	L	37,86	L
31		LL	24,54	L	26,96	L	17,76	L	26,12	L
34		LL	24,68	L	27,04	L	18,18	L	27,22	L
36		LL	30,61	L	28,71	L	17,61	L	30,33	L

Summary of the samples tested for each PCR system from MB patients

**Clinical Form**	**Total number**	**sodA(%) positivity**	**16S (%) positivity**	**RLEP (%) positivity**	**85B (%) positivity**
**BB**	5	4 (80)	5 (100)	5 (100)	5 (100)
**BL**	5	5 (100)	5 (100)	5 (100)	5 (100)
**LL**	11	11 (100)	11 (100)	11 (100)	10 (90.9)

Paucibacillary patients

Biopsy Number	Observations	Clinical Form	Ct 16S rRNA	Pred. Class 16S	Ct 85B	Pred. Class 85B	Ct RLEP	Pred. Class RLEP	Ct sodA	Pred. Class sodA
46	RR	BT	NA	NL	NA	NL	39,00	L	NA	NL
87		BT	37,98	L	35,46	L	29,91	L	36,71	L
88		BT	34,84	L	34,26	L	16,91	L	34,31	L

89		BT	NA	NL	NA	NL	31,35	L	NA	NL
91		BT	NA	NL	NA	NL	34,74	NL	NA	NL
92		BT	39,03	NL	38,69	L	NA	NL	NA	NL
94		BT	NA	NL	NA	NL	NA	NL	NA	NL
95		BT	NA	NL	39,43	L	33,89	NL	NA	NL
97		BT	NA	NL	NA	NL	35,68	L	NA	NL
98		BT	NA	NL	NA	NL	34,64	NL	NA	NL
67*		BT	NA	NL	NA	NL	35,21	L	NA	NL
3		I	NA	NL	NA	NL	34,25	NL	NA	NL
8*		I	NA	NL	NA	NL	35,24	NL	NA	NL
70		I	NA	NL	NA	NL	39,51	L	NA	NL
71		I	NA	NL	38,92	L	37,49	L	NA	NL
72		I	NA	NL	NA	NL	35,70	L	NA	NL
73		I	NA	NL	NA	NL	37,38	L	NA	NL
74		I	34,00	L	36,38	L	27,99	L	NA	NL
75		I	NA	NL	NA	NL	37,39	L	NA	NL
76		I	NA	NL	NA	NL	39,51	L	NA	NL

77		I	NA	NL	NA	NL	35,95	L	NA	NL
78		I	NA	NL	NA	NL	NA	NL	NA	NL
29*		I	38,70	NL	NA	NL	38,56	L	NA	NL
43	Biopsy from healthy skin	NP	NA	NL	NA	NL	39,52	NL	NA	NL
93		TT	NA	NL	NA	NL	37,75	L	NA	NL
96		TT	NA	NL	NA	NL	35,49	L	NA	NL

Summary of the samples tested for each PCR system from PB patients

Clinical form	Total number	sodA(%) positivity	16S (%) positivity	RLEP (%) positivity	85B (%) positivity
**BT**	11	2 (18.2)	2 (18.2)	6 (54.5)	4 (36.4)
**I**	12	0	1 (8.3)	9 (75)	2 (16.7)
**NP/TT**	3	0	0	2 (66.7)	0

* These patients were initially classified as controls, but confirmation of leprosy was done only after databank search (5-10 years after biopsy collection). Then, biospies were reanalyzed histologically and also reclassified as patients according to R&J classification.

Non-leprosy patients and normal individuals

Biopsy Number	Observations	Clinical Form	Ct 16S rRNA	Pred. Class 16S	Ct 85B	Pred. Class 85B	Ct RLEP	Pred. Class RLEP	Ct sodA	Pred. Class sodA
12	Capillaritis	NL	38,43	NL	NA	NL	34,14	L	39,04	NL
17	Cutaneous Mucinosis	NL	NA	NL	NA	NL	34,76	L	NA	NL
38	Chronic Dermatitis	NL	38,27	NL	NA	NL	32,01	L	NA	NL
41	Normal skin (healthy person)	NL	NA	NL	NA	NL	NA	NL	NA	NL

45	Normal skin (healthy person)	NL	NA	NL	NA	NL	NA	NL	NA	NL
47	Normal skin (healthy person)	NL	NA	NL	NA	NL	39,52	NL	NA	NL
49	Normal skin (healthy person)	NL	NA	NL	NA	NL	NA	NL	NA	NL
50	Normal skin (healthy person)	NL	NA	NL	NA	NL	39,85	NL	NA	NL
61	Indeterminate Chronic Dermatitis	NL	NA	NL	NA	NL	NA	NL	NA	NL
62	Erythema Dyschromicum	NL	NA	NL	NA	NL	NA	NL	NA	NL
64	Indeterminate Chronic Dermatitis	NL	38,07	NL	NA	NL	37,87	L	NA	NL
65	Reticular Erythematous Mucinosis	NL	NA	NL	NA	NL	NA	NL	NA	NL
66	Indeterminate Chronic Dermatitis	NL	NA	NL	NA	NL	NA	NL	NA	NL
68	Non Leprotic Polymorphic Erythema	NL	NA	NL	NA	NL	NA	NL	NA	NL
69	Chronic Eczema	NL	NA	NL	NA	NL	39,89	NL	NA	NL

Summary of the samples tested for each PCR system from non-leprosy patients and normal individuals

**Clinical Form**	**Number**	**sodA(%) positivity**	**16S (%) positivity**	**RLEP (%) positivity**	**85B (%) positivity**
**Skin from healthy donor**	5	0	0	0	0
**Other dermatological diseases**	10	0	0	4	0
